# ATRX, OLIG2, MGMT, and IDH2 in Glioblastoma: Essential Molecular Mechanisms and Therapeutic Significance

**DOI:** 10.3390/medicina61040697

**Published:** 2025-04-10

**Authors:** Andrea Pop-Crisan, Radu Pirlog, Lavinia-Lorena Pruteanu, Constantin Busuioc, Ovidiu-Laurean Pop, Deo Prakash Pandey, Cornelia Braicu, Ioana Berindan-Neagoe

**Affiliations:** 1 Department of Surgical Sciences, Faculty of Medicine and Pharmacy, University of Oradea, 410073 Oradea, Romania; andrea20_crisan@yahoo.com (A.P.-C.); popo@uoradea.ro (O.-L.P.); 2 Department of Genomics, MEDFUTURE Institute for Biomedical Research, Iuliu Hațieganu University of Medicine and Pharmacy, 400347 Cluj-Napoca, Romania; radupirlogmg@gmail.com (R.P.); pruteanulavinia@gmail.com (L.-L.P.); 3 Département de Pathologie, Hôpitaux Universitaires Henri Mondor, AP-HP, 94010 Créteil, France; 4 INSERM U955, Université Paris Est Créteil, 94010 Créteil, France; 5 Department of Chemistry and Biology, North University Center, Technical University of Cluj-Napoca, 430122 Baia Mare, Romania; 6 Department of Pathology, Spitalul Clinic Sfanta Maria, Bulevardul Ion Mihalache 37-39, 011172 București, Romania; busuioc.constantin@gmail.com; 7 Department of Pathology, Onco Team Diagnostic, 010719 Bucharest, Romania; 8 Centre for Embryology and Healthy Development, Department of Microbiology, Rikshospitalet, Oslo University Hospital, 0372 Oslo, Norway; deo.prakash@gmail.com; 9 Doctoral School Iuliu Haţieganu, University of Medicine and Pharmacy, 400347 Cluj-Napoca, Romania; ioana.neagoe@umfcluj.ro; 10 Biomedical Sciences Sections, Romanian Academy of Medical Sciences, 030167 București, Romania

**Keywords:** glioblastoma, molecular alteration, therapeutic strategy

## Abstract

*Background and Objectives*: Glioblastoma (GBM) is among the most aggressive and lethal primary brain tumors, characterized by high heterogeneity, invasive growth, and resistance to conventional therapies. The 2021 WHO classification highlights the importance of molecular diagnostics, integrating genetic, transcriptomic, and epigenetic alterations alongside histological and immunohistochemical criteria. *Materials and methods*: Key molecular regulators, including ATRX, OLIG2, MGMT, and IDH2, play critical roles in chromatin remodeling, transcriptional reprogramming, DNA repair, and metabolic adaptation. However, their specific expression patterns and functional roles in GBM remain incompletely understood. This study utilizes publicly available data from The Cancer Genome Atlas (TCGA) to assess the transcriptional profiles of ATRX, OLIG2, MGMT, and IDH2 in GBM, aiming to identify potential biomarkers and therapeutic targets. *Results*: The expression analysis revealed that ATRX is downregulated at the gene level but overexpressed at the protein level, while OLIG2 is consistently overexpressed at both levels. MGMT showed no statistically significant changes in either gene or protein expression, whereas IDH2 was not significantly altered at the gene level but was downregulated at the protein level (*p* < 0.05). These discrepancies suggest potential post-transcriptional regulatory mechanisms influencing GBM molecular profiles. Notably, OLIG2 and MGMT expression correlated significantly with patient survival (*p* < 0.05), whereas ATRX and IDH2 did not reach statistical significance. *Conclusions*: Understanding these molecular relationships provides valuable insights into potential therapeutic strategies, paving the way for precision oncology approaches and combination therapies targeting multiple pathways simultaneously.

## 1. Introduction

Glioblastoma (GBM) is the most aggressive and lethal primary brain tumor in adults, being the most frequent type of glioma [1]. Despite many advances in diagnostic imaging, surgical resection, radiation therapy, and chemotherapy, the prognosis for GBM remains dismal, with a median survival time of approximately 15 months following standard treatment represented by surgical resection followed by radiotherapy and chemotherapy with Temozolomide (TMZ) [1,2]. TMZ is a DNA alkylating agent, and while it is considered well-tolerated, treatment with TMZ extends survival benefits only by a few months with substantial side effects. This poor outcome is attributed to the intrinsic heterogeneity, invasive growth, rapid proliferation, high recurrence, and resistance to conventional therapies [3,4].

GBM is categorized into molecular subtypes based on several genetic, transcriptomic, and epigenetic alterations, as outlined in the 2021 World Health Organization (WHO) classification of central nervous system (CNS) tumors [5]. This edition marks a pivotal shift, emphasizing the role of molecular diagnostics in CNS tumor classification while integrating traditional diagnostic methods, such as histology and immunohistochemistry (IHC). The updated classification introduces new molecular approaches to CNS tumor nomenclature and grading, emphasizing the importance of integrated diagnoses and layered reporting [4,5,6].

Therapeutic targeting of GBM remains challenging due to the tumor’s dynamic adaptability under external stress, which renders standard treatment strategies insufficient [1,7,8]. The pathogenesis of GBM involves many genetic and epigenetic alterations, including mutations, chromosomal rearrangements, and activation of aberrant signalling pathways, which collectively drive tumor initiation, progression, and therapeutic resistance [1,8].

Key molecular players, such as ATRX, OLIG2, MGMT, and IDH2 proteins, contribute to critical oncogenic processes like chromatin remodelling, glioblastoma cell fate and de-differentiation, DNA repair and metabolic reprogramming. Understanding the roles of these molecular players in GBM pathogenesis enhances our understanding of tumor biology. It highlights potential therapeutic targets, paving the way for novel strategies to overcome the challenges of this aggressive malignancy. ATRX is a chromatin remodelling factor that is frequently altered in glioblastoma patients and is associated with maintenance defects. OLIG2, a neurodevelopmental transcription factor, is crucial for neural development and plays a key role in driving a transcriptional program that sustains the proliferation of glioblastoma stem cells. MGMT (O6-methylguanine-DNA methyltransferase) is involved in DNA repair mechanisms and is a known determinant of resistance to alkylating agents such as TMZ, the standard chemotherapeutic drug for GBM. Evaluating the methylation status of the promoter of the MGMT gene is a standard diagnostic practice in GBM and needs to be routinely evaluated in all neuropathology laboratories [9,10,11]. IDH2, an isoform of isocitrate dehydrogenase, plays a role in metabolic reprogramming and oncometabolite production in glioblastoma. Mutations in IDH2 (e.g., R172K) lead to the accumulation of D-2-hydroxyglutarate (D-2HG), which disrupts epigenetic regulation and alters metabolism. However, due to the rarity of IDH2 mutations in GBM, their clinical and prognostic impact remains poorly understood.

ATRX, OLIG2, MGMT, and IDH2 were selected due to their critical roles in glioblastoma biology and prognosis. Despite extensive research, the expression patterns of these genes in GBM versus normal brain tissue remain partially understood. This study utilizes TCGA datasets to analyze their transcriptional profiles, aiming to clarify their roles in GBM pathophysiology and their potential therapeutic relevance.

## 2. Materials and Methods

**Haematoxylin and eosin (H&E) staining.** H&E staining was done on GBM samples using standard automatic histologic strainers on 5 um sections. Two experienced pathologists (OP, RP) analysed HE slides to identify representative diagnostic features of GBM. This study was conducted in accordance with ethical standards and received approval from the Institutional Ethics Committee of Clinical Hospital Oradea (Approval No. 38801/18.11.22).

**Immunohistochemistry (IHC).** Immunohistochemical analysis was performed using automated stainers and recommended protocols, with antibodies targeting OLIG2, MGMT, IDH2, and Ki-67 on glioblastoma formalin-fixed paraffin-embedded (FFPE) tissue sections. These four markers are nuclear markers. A moderate or intense nuclear staining was considered positive. IHC was interpreted on a dichotomous scale (positive/negative) for OLIG2, MGMT, and IDH2. Ki-67 expression was quantitatively assessed as the percentage of positively stained tumor cells. IHC slides were independently evaluated by two experienced pathologists.

**Gene and protein expression analysis in GBM.** UALCAN (http://ualcan.path.uab.edu/, 23 January 2025) is an interactive web resource that facilitates the analysis of gene expression data from The Cancer Genome Atlas (TCGA) [12]. This study utilized UALCAN to assess the expression levels of ATRX, OLIG2, MGMT, and IDH2 in GBM samples. The dataset included 156 primary tumor samples and 5 normal brain samples for comparative analysis. UALCAN provides access to TCGA transcriptomic data, allowing for visualization and statistical gene and protein expression analysis across different tumor types and subgroups, including GBM [13]. The platform also enables comparisons between tumor and normal tissues, assessment of promoter methylation levels, and correlation analysis with clinicopathological parameters. UALCAN’s inbuilt analytical tools determined statistical significance, facilitating the identification of potential biomarkers [12,13]. Kaplan–Meier survival analysis was performed using UALCAN, based on TCGA data, via the log-rank test to compare survival distributions.

For the pan-cancer overview, we utilized TIMER2.0 (http://cistrome.org/TIMER/, 24 January 2025) to analyze the expression of ATRX, OLIG2, MGMT, and IDH2 across TCGA cancers. The “Gene Expression” module was used to compare tumor and normal tissue expression in **log2(TPM+1)** format, applying **Wilcoxon rank-sum tests** for significance. TIMER2’s built-in boxplots visualized expression differences, with statistical significance indicated by asterisks [14]. This analysis provided insights into the broader dysregulation of these genes beyond different cancer types.

**STRING Database Analysis.** The STRING database (https://string-db.org/, 28 January 2025) was used to explore the functional interactions among ATRX, OLIG2, MGMT, and IDH2. The constructed interaction (PPI) network was analyzed to determine key hubs and connectivity patterns, and visualization was performed using STRING’s built-in graphical tools. Visualization was performed using STRING’s built-in graphical tools, allowing for an intuitive representation of potential direct and indirect associations.

## 3. Results

**Figure 1:**
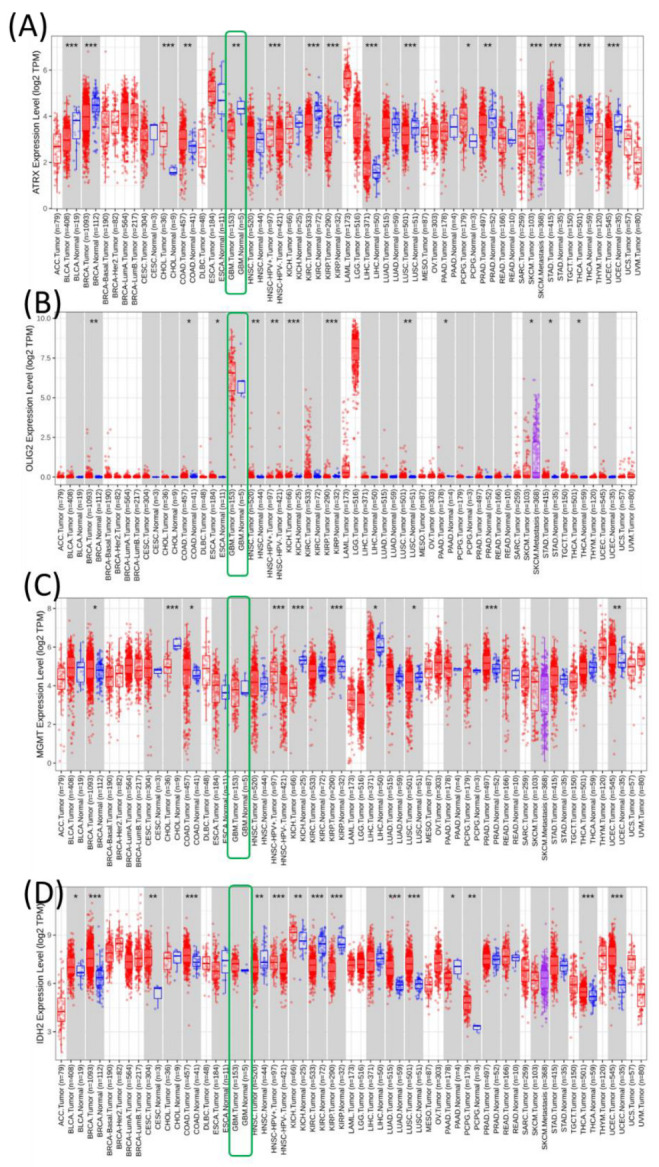
Pan-cancer expression analysis of ATRX, OLIG2, MGMT, and IDH2 across various tumor types from the TCGA dataset. (**A**) ATRX expression levels; (**B**) OLIG2 expression levels; (**C**) MGMT expression levels; (**D**) IDH2 expression levels. Expression is represented as log2 TPM (Transcripts Per Million) values for tumor samples (red) and normal tissue samples (blue). Green Box- highlights the expression level in GBM for the selected genes. The statistical significance of differential expression is as follows: * *p* < 0.05, ** *p* < 0.01, *** *p* < 0.001. Tumor types are annotated on the *x*-axis, with the number of samples indicated in parentheses.

**Pan-cancer overview of the expression of ATRX, OLIG2, MGMT, and IDH2 genes.** These genes were analyzed using the TCGA dataset downloaded from TIMER2 to create pan-cancer expression profiles across various cancer types. Each subpanel corresponds to one gene (ATRX, OLIG2, MGMT, and IDH2) and displays its expression levels in tumors (red) and normal tissues (blue) across multiple cancer types (Figure 1).

ATRX expression is significantly upregulated in several cancer types, including ACC, GBM, lower-grade gliomas (LGG), and pancreatic adenocarcinoma (PAAD), compared to their normal counterparts. However, the expression levels in cancers such as KIRC and lung adenocarcinoma (LUAD) show contrasting patterns. OLIG2 is primarily expressed in central nervous system-related cancers, such as GBM and LGG, where it is significantly upregulated. Expression is minimal or absent in most other cancer types, reflecting its role as a neural-specific transcription factor. MGMT expression exhibits variable patterns. It is significantly upregulated in certain cancers like GBM and LGG, while showing no substantial difference or downregulation in others, such as PRAD and KICH. This variability might reflect the differential need for DNA repair mechanisms across tumor types. IDH2 is ubiquitously expressed across many cancers, with significant upregulation observed in tumors like ACC, LGG, and GBM. The high expression in these cancers aligns with its role in metabolic reprogramming in tumor cells.

**Figure 2:**
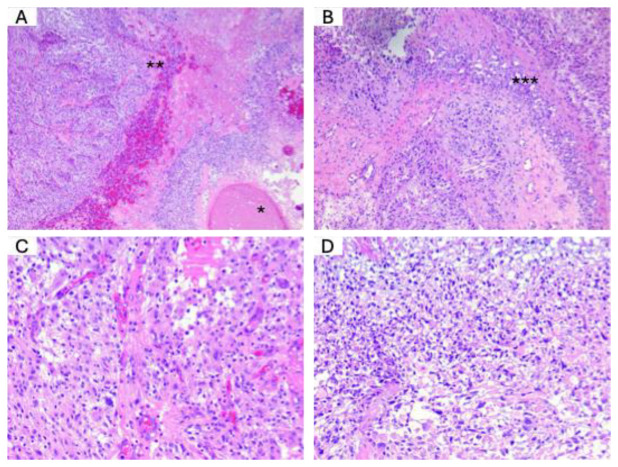
Haematoxylin and eosin staining in GBM. (**A**) The primary histological characteristics of glioblastoma include intratumoral necrosis (*), vascular proliferation, and diffuse tumor infiltration of normal brain tissue (**). Haematoxylin–eosin, 40×. (**B**) The presence of peripheral palisading (***-intratumoral necrosis), a distinctive histological feature of glioblastoma, along with multiple intratumoral vessels, demonstrates the abundant vascularisation of this aggressive tumor. Haematoxylin–eosin, 100×. (**C**,**D**) A high-magnification view of glioblastoma cells reveals a pleomorphic proliferation of tumor cells with atypical cells that possess large, pleomorphic nuclei and multiple mitoses. (**D**) It displays a variant of glioblastoma characterised by clear cytoplasm and atypical cells exhibiting significant size variations and nuclear pleomorphism; the size variation of tumor cells exceeds three times that of a normal brain cell. Haematoxylin–eosin 400×.

**H&E staining.** Next, we performed H&E staining to assess the histological characteristics of the tumor samples. The histopathological diagnosis was conducted following the latest edition of the WHO 2021 classification. This classification provides standardized criteria for diagnosing and grading CNS tumors based on morphological features, aiding in accurate classification and clinical decision-making. Figure 2 illustrates the key histological features observed in a typical glioblastoma.

**Figure 3:**
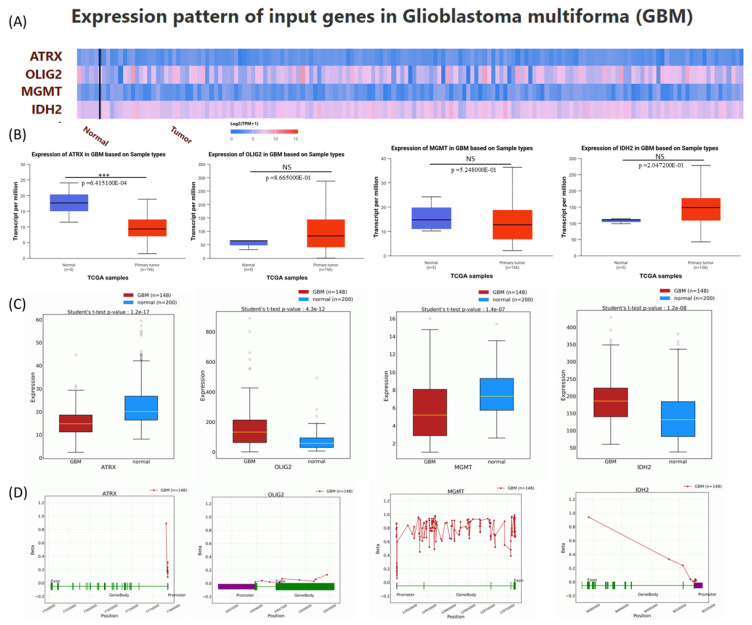
Expression analysis of the selected key molecular players in glioblastoma (GBM) using UALCAN and OncoBD. (**A**) A heatmap was generated to depict ATRX, OLIG2, MGMT, and IDH2 expression profiles in normal and tumor tissue downloaded from UALCAN. (**B**) The expression levels are represented as boxplots according to UALCAN. (**C**) The expression levels are represented as boxplots according to OncoBD. (**D**) The methylation status according to OncoBD. Significant upregulation or downregulation of these genes in tumor samples compared to normal tissues is highlighted, with *p*-values indicating statistical significance (*** *p* ≤ 0.001).

**Expression analysis of gene expression patterns for ATRX, OLIG2, MGMT, and IDH2 in GBM.** The expression levels of these genes are obtained from the UALCAN database, which is based on data derived from the TCGA database, to provide novel insights into their potential roles in GBM pathogenesis [12,13]. A heatmap was generated to depict the expression profiles of ATRX, OLIG2, MGMT, and IDH2 in normal and tumor tissues, using Log2(TPM+1) for scaling (Figure 3A). Boxplots were employed to statistically assess expression differences, with *p*-values calculated using the Student’s *t*-test. Non-significant results (NS) were defined as *p* > 0.05. Expression levels for ATRX were significantly reduced in GBM tumors compared to normal tissues. No significant differences in expression were observed between normal and GBM samples for OLIG2, MGM, and IDH2 (Figure 3B).

Additionally, gene expression levels from the OncoDB database were presented, showing consistency with data from UALCAN, except for MGMT, which exhibited downregulation in tumor tissues (Figure 3C). This discrepancy may be explained by differences in sample size and the high heterogeneity of the disease. In Figure 3D, the methylation pattern is presented with data downloaded from the OncoBD database.

**Figure 4:**
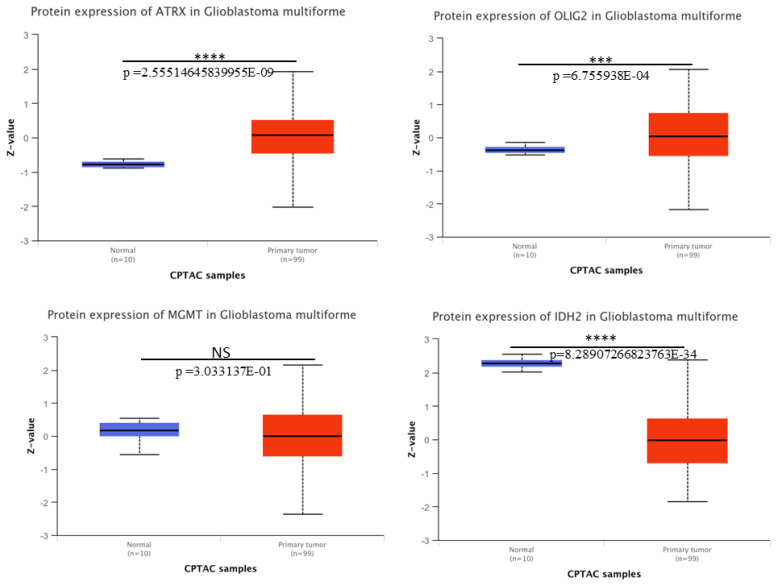
Protein expression analysis of the selected key molecular players in GBM using UALCAN. The expression levels are represented as boxplots. Significant upregulation or downregulation of these genes in tumor samples compared to normal tissues is highlighted, with *p*-values indicating statistical significance.

**Protein expression patterns for ATRX, OLIG2, MGMT, and IDH2 in GBM.** We downloaded the protein expression of these key molecular markers in GBM using CPTAC from UALCAN. Boxplots depict the protein expression levels (Z-values) of ATRX, OLIG2, MGMT, and IDH2 in normal tissue samples (n = 10, blue) and primary tumor samples (n = 99, red). A significant upregulation of ATRX, OLIG2, and downregulation of IDH2 at protein level was observed in GBM tumor samples compared to normal tissues (Figure 4), with *p*-values indicating statistical significance (**** *p* < 0.0001; *** *p* < 0.001). MGMT shows no significant difference (NS, *p* = 0.303) between normal and tumor tissues. The analysis highlights the differential expression patterns of these markers, thereby contributing to an understanding of GBM pathogenesis.

**Figure 5:**
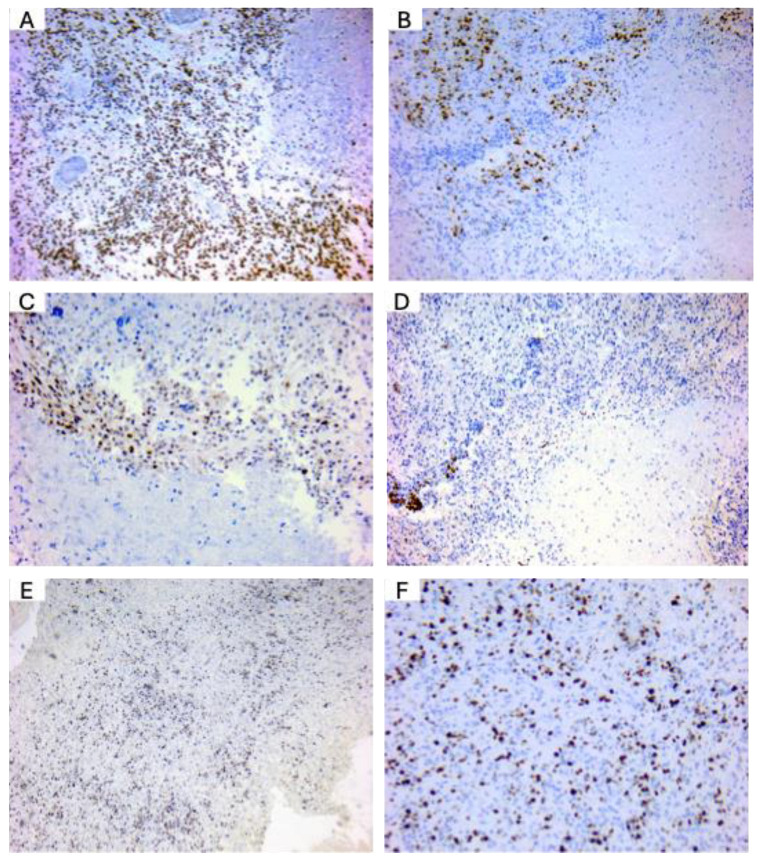
Immunohistochemical evaluation of glioblastoma using standard diagnostic markers. (**A**) ATRX, 100×. Positive nuclear cells. (**B**) Olig2, 100×. Positive cells show a nuclear staining. (**C**) MGMT, 100×. MGMT-negative expression in glioblastoma wild-type in the nuclei of tumor cells. (**D**) IDH2-132H, 100×. The image shows pseudo-palisading necrosis with wild-type IDH 1/2 expression. In gliomas, including glioblastoma, mutations in the IDH1 gene typically involve a specific mutation at the arginine residue 132 (R132), most commonly an R132H substitution. (**E**,**F**) Ki-67—marker of proliferation, showing a high proliferation index in the tumor cells at low (**E**) and high (**F**) magnification. Ki-67 is a nuclear marker that also allows the morphological analysis of tumor nuclei and identification of atypia.

**OLIG2 and MGMT are well-expressed in glioblastoma patients.** To confirm the expression of key diagnostic markers in glioblastoma (GBM), we performed immunohistochemical staining on GBM tissue samples obtained from diagnostic cases in our department. Representative images are presented in Figure 5. ATRX expression was observed as positive nuclear staining in tumor cells, consistent with its role in chromatin remodeling and indicating the presence of ATRX protein in the analyzed glioblastoma samples (Figure 5A). Olig2 expression was observed in the nuclei of tumor cells, with intense positive staining particularly prominent in perinecrotic regions. This pattern aligns with Olig2’s role as a key transcription factor associated with glioblastoma stem-like cells (Figure 5B). MGMT expression analyses showed MGMT-negative tumor cells, indicative of MGMT promoter methylation in these glioblastoma cases (Figure 5C). IDH2-132H staining did not reveal evidence of mutant IDH1/2 expression, supporting a diagnosis of IDH-wild-type glioblastoma (Figure 5D). Ki-67 staining (Figure 5E,F, low and high magnification, respectively) revealed a high proliferation index, a hallmark of glioblastoma. Ki-67, a nuclear proliferation marker, facilitates morphological evaluation of tumor nuclei and aids in identifying cellular atypia, which is frequently observed in GBM. These findings confirm the expression patterns of standard glioblastoma markers and support the histopathological diagnosis based on the WHO Classification of Central Nervous System Tumors.

**Figure 6:**
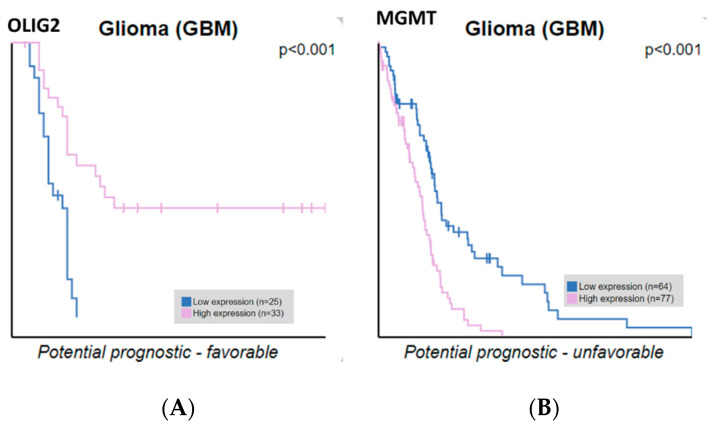
Kaplan–Meier survival curves for GBM patients, for OLIG2 and MGMT protein expression levels, downloaded from the Human Protein Atlas database. (**A**) OLIG2 Kaplan–Meier survival curves indicate that high OLIG2 expression (pink line) correlates with improved prognosis, suggesting a potential tumor-suppressive role or association with less aggressive glioma subtypes. (**B**) MGMT Kaplan–Meier survival curves indicate that high MGMT expression (pink line) is linked to a worse prognosis, likely due to its role in DNA repair and resistance to alkylating chemotherapy, such as temozolomide. The statistical significance (*p* < 0.001) suggests a strong association between gene expression levels and patient survival outcomes.

**ATRX, OLIG2, MGMT, and IDH2 expression correlate with GBM patient survival.** We extracted ATRX, OLIG2, MGMT, and IDH2 expression data and correlated them with the survival of glioblastoma patients. Our findings indicate that OLIG2 and MGMT expression levels are significantly correlated with overall survival, whereas ATRX and IDH2 do not reach statistical significance. Only OLIG2 and MGMT showed statistically significant correlations with overall survival, whereas ATRX and IDH2 did not reach statistical significance, as analyzed from the Human Protein Atlas (Figure 6). The Kaplan–Meier curves compare patient survival between groups with high (pink line) and low (blue line) gene expression. OLIG2 upregulation is associated with more prolonged survival, potentially reflecting its role in maintaining a less aggressive, oligodendrocyte-like glioma phenotype. Conversely, high MGMT expression correlates with shorter survival, emphasizing its role in chemotherapy resistance by repairing DNA damage induced by alkylating agents. These results underscore the prognostic value of OLIG2 and MGMT in glioblastoma, supporting their potential utility in guiding therapeutic strategies. These results highlight the importance of molecular stratification in refining prognostic assessments and guiding personalized therapeutic strategies in glioblastoma.

**Figure 7:**
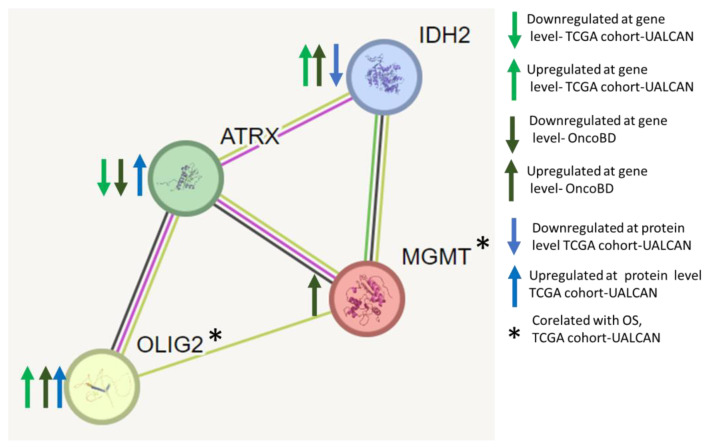
STRING Network Analysis illustrates the interactions between ATRX, OLIG2, MGMT, and IDH2. This STRING network visualisation illustrates the predicted and experimental evidence interactions among ATRX, OLIG2, MGMT, and IDH2, key molecular markers implicated in glioma biology. Nodes represent proteins, while edges indicate functional and physical interactions (blue arrow: gene expression alteration GBM, green arrow: expression alteration GBM, * protein correlated with overall survival rate in GBM).

**Protein–protein interaction network analyses of ATRX, OLIG2, MGMT, and IDH2 identify them as key nodes in regulating GBM.** We performed protein–protein interaction network analyses using the STRING database and found that ATRX, OLIG2, MGMT, and IDH2 interact (Figure 7). Each node represents a protein, and edges indicate known or predicted functional associations. The interactions are inferred from various sources, including curated databases, experimental data, and computational predictions.

**Figure 8:**
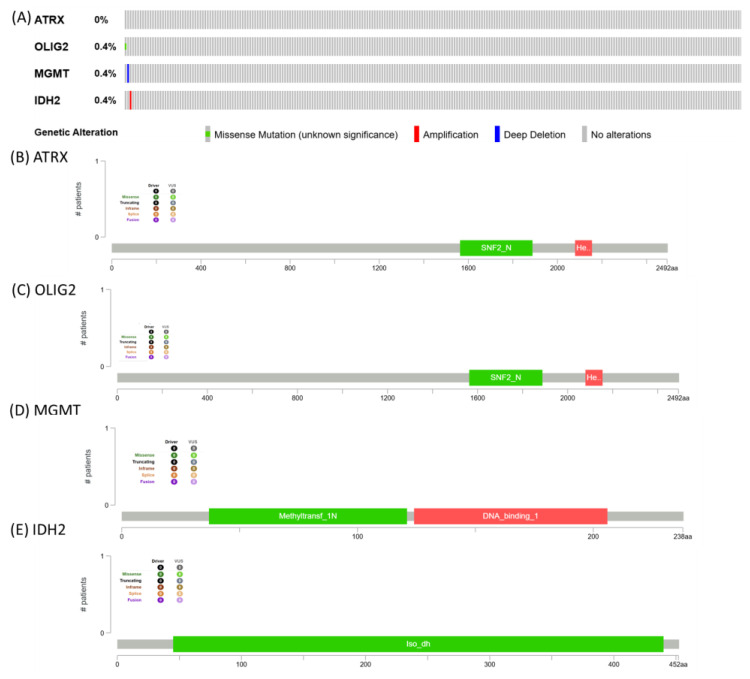
The frequency and types of genetic alterations for ATRX, OLIG2, MGMT, and IDH2. (**A**) Heatmap graphical representation of mutation frequency for ATRX, OLIG2, MGMT, and IDH2, downloaded from cBioPortal (https://www.cbioportal.org, accessed on 12 February 2025). The “lollipop” plot was generated by the MutationMapper tool of cBioPortal for (**B**) ATRX gene, (**C**) OLIG2 gene, (**D**) MGMT gene, and (**E**) IDH2 gene.

**Mutational pattern for ATRX, OLIG2, MGMT, and IDH2**. The image presents genomic alteration data for ATRX, OLIG2, MGMT, and IDH2, summarizing mutation frequencies and protein domain structures. ATRX shows no alterations, while OLIG2, MGMT, and IDH2 each exhibit a 0.4% alteration frequency, with genetic changes including missense mutations (green), amplifications (red), and deep deletions (blue) (Figure 8).

## 4. Discussion

ATRX, OLIG2, MGMT, and IDH2 expression profiles in GBM reveal a complex interplay between gene and protein levels. While gene-level expression data (transcriptional analysis) from databases such as TCGA often indicate differential regulation of these key molecular markers, the protein-level expression data presented here (derived from CPTAC, UALCAN) demonstrate nuanced discrepancies.

The low mutation frequency observed in ATRX, OLIG2, MGMT, and IDH2 suggests that genetic alterations in these genes may not be a primary driver in the TCGA cohort. This finding is consistent with the idea that glioblastoma and other aggressive tumors can exhibit heterogeneity, with alterations occurring at different molecular levels, such as epigenetic modifications, transcriptomic dysregulation, or post-translational modifications, rather than frequent somatic mutations [15,16,17]. In particular, the absence of ATRX mutations contrasts with its well-documented role in lower-grade gliomas and astrocytomas, implying that alternative mechanisms, such as chromatin remodeling defects, might be involved in tumor progression [18]. Similarly, the low mutation rate in MGMT may suggest that its expression and promoter methylation status, rather than direct genetic alterations, play a more significant role in treatment response and tumor biology [19]. The minimal alterations in IDH2 align with previous findings, indicating that IDH mutations are more commonly associated with lower-grade gliomas rather than glioblastoma [18,20]. Overall, these results underscore the need for a more comprehensive, integrative approach that incorporates both transcriptomic and epigenomic analyses to gain a deeper understanding of the molecular landscape of these tumors [17].

Proteins are responsible for cellular degradation processes, such as ubiquitination and proteasomal activity [21]. Enhanced degradation could explain why some markers show reduced or unchanged protein levels despite elevated gene expression. Conversely, stabilization of proteins through post-translational modifications could lead to higher protein levels independent of mRNA abundance [22], as observed for markers like ATRX and IDH2.

ATRX mRNA levels, as depicted in pan-cancer analysis, are significantly upregulated in GBM compared to normal tissues [23]. This upregulation aligns with ATRX’s role in chromatin remodeling and transcriptional regulation, which can promote tumorigenesis in specific contexts. ATRX dysfunction is commonly associated with glioma mutations, particularly in lower-grade gliomas and secondary GBM, and is often linked to altered telomere maintenance mechanisms [23]. In contrast to the high mRNA levels, protein expression analysis using CPTAC data reveals a different picture. ATRX protein levels are also significantly elevated in GBM tumor tissues compared to normal controls, supporting the transcriptional data. However, the extent of protein elevation may not directly correlate with mRNA upregulation, suggesting additional layers of regulation at the post-transcriptional or post-translational levels. This dual-level analysis highlights the complexity of ATRX regulation in GBM and underscores the need to comprehensively integrate transcriptomic and proteomic data to understand its role in tumorigenesis. ATRX, IDH1/2, and Ki-67 collaboratively define three distinct subgroups of astrocytic tumors, offering a molecular classification that transcends the conventional WHO grading system [24]. ATRX knockout has been shown to suppress malignant behaviors in glioma cells and plays a critical role in regulating DNA damage repair through the ATM signaling pathway.

Additionally, ATRX expression is associated with the chemosensitivity of GBM patients, highlighting its potential as a therapeutic target [25,26]. A growing body of evidence indicates that tumor propagation and resistance to therapy are driven by a subset of stem-like cells, which rely on core developmental transcription factors (TFs) to maintain their specific transcriptional programs and sustain proliferation [27,28]. 

Among TF-Sox2, Sall2, Pou3f2, and notably, Olig2, Olig2 emerged as a key developmental regulator with well-established roles in neural progenitor differentiation during normal brain development. In the context of GBM, Olig2 contributes to the maintenance of tumor-initiating cells and regulates pathways that promote cell survival and proliferation. However, emerging data challenges the assumption that these core TFs are uniformly expressed across all GBM cells. Reports imply that the expression of Olig2 and Sall2 is variable among GBM patient samples [29]. This heterogeneity in Olig2 expression raises important questions about its precise role in GBM biology. It suggests that while Olig2 may be critical for developing and maintaining specific GBM cell subpopulations, its function and regulatory impact might differ across tumors. Our findings underscore the significance of Olig2 as a central developmental regulator in GBM, highlighting the need for further studies to elucidate its role in tumor heterogeneity. OLIG2 has also been implicated in glioma biology, with its upregulation potentially maintaining a less aggressive tumor phenotype and contributing to better outcomes [30].

Decreased protein expression of IDH2 may lead to impaired NADPH production, resulting in an imbalance in redox homeostasis and increased susceptibility to oxidative damage. Such alterations could promote the survival of tumor cells under hypoxic conditions, a hallmark of GBM pathophysiology. Moreover, the loss of α-KG production may disrupt cellular metabolism and epigenetic regulation, as α-KG is a co-factor for dioxygenase enzymes, including those involved in DNA and histone demethylation [31,32]. Notably, mutations in IDH1 and IDH2 are well-characterized in lower-grade gliomas and secondary GBM, leading to the production of the oncometabolite 2-hydroxyglutarate (2-HG), which contributes to epigenetic dysregulation and tumorigenesis. However, IDH2 mutations are rare in primary GBM. IDH2 downregulation at the protein level without mutation suggests that mechanisms other than direct genetic alterations, such as proteasomal degradation or microRNA-mediated suppression, may influence its expression [31]. Therapeutically, the downregulation of IDH2 may present challenges, as restoring its function could potentially enhance the redox capacity of tumor cells [31,33]. However, targeting downstream metabolic vulnerabilities associated with IDH2 deficiency, such as disrupted mitochondrial metabolism or increased oxidative stress, could offer novel therapeutic opportunities [33]. Further studies are warranted to elucidate the exact mechanisms underlying IDH2 downregulation at the protein level and its functional implications in GBM [31].

Low or absent MGMT expression in glioblastoma cells can be used as a surrogate marker for the hypermethylation of the MGMT gene promoter [34]. MGMT promoter methylation is detected in approximately 35–45% of glioblastomas. This epigenetic modification is a predictive biomarker of better response to temozolomide (TMZ) chemotherapy and a favorable prognostic factor, as patients with methylated MGMT promoters typically exhibit longer overall survival than those with unmethylated promoters [19,35]. According to the WHO 2021 recommendation, the MGMT methylation status should be performed on all newly diagnosed GBM [5]. Recently, Long et al. highlighted a new therapeutic approach using a triplet of immune checkpoint blockade in newly diagnosed GBM, where the MGMT status might play an essential role in the response to this new combined therapeutic approach [36].

The network presented in Figure 7 provides insights into the molecular mechanisms underlying glioma progression, particularly alterations in chromatin remodeling (ATRX), neurodevelopmental transcriptional regulation (OLIG2), DNA repair (MGMT), and metabolic reprogramming (IDH2). The edge thickness corresponds to the confidence level of each interaction, reflecting the strength of the predicted association.

This network analysis reinforces that gliomas are driven by complex molecular interactions rather than single-gene alterations. Understanding these interactions is crucial for identifying vulnerabilities that can be exploited for therapeutic intervention. Future research should focus on integrating multi-omics data to elucidate these regulatory networks further and refine precision medicine strategies for GBM treatment.

## 5. Conclusions

The ATRX, OLIG2, MGMT, and IDH2 networks highlight key molecular interactions that contribute to glioma development, progression, and therapeutic resistance. ATRX loss and OLIG2 overexpression support glioma stemness and chromatin remodeling, while MGMT activity influences chemoresistance. IDH2 mutations drive metabolic reprogramming, further shaping the tumor microenvironment. The observed regulatory changes at both transcriptomic and proteomic levels underscore the complexity of these interactions and the need for multi-omics approaches in glioma research. The observed discrepancies between gene and protein expression levels highlight the need for integrated multi-omics analyses to elucidate the complex regulatory mechanisms in GBM. Understanding these discrepancies is critical for accurately identifying actionable molecular targets and developing effective therapeutic strategies for this aggressive malignancy.

Understanding these molecular relationships provides valuable insights into potential therapeutic strategies from a clinical perspective. For example, ATRX-deficient gliomas may benefit from telomerase-targeting therapies, OLIG2 inhibition could improve glioblastoma treatment response, MGMT promoter methylation remains a key predictive biomarker for temozolomide efficacy, and IDH-mutant gliomas show promise for targeted metabolic interventions.

Future research should focus on integrating these molecular insights into precision oncology approaches, refining diagnostic markers, and developing combination therapies that target multiple pathways simultaneously. From a systems biology standpoint, incorporating transcriptomic, proteomic, and metabolomic data will be crucial for unravelling glioma heterogeneity and identifying novel therapeutic vulnerabilities.

## Data Availability

Available on request to the corresponding author.
